# Testing the PEST hypothesis using relevant Rett mutations in MeCP2 E1 and E2 isoforms

**DOI:** 10.1093/hmg/ddae119

**Published:** 2024-08-14

**Authors:** Ladan Kalani, Bo-Hyun Kim, Alberto Ruiz de Chavez, Anastasia Roemer, Anna Mikhailov, Jonathan K Merritt, Katrina V Good, Robert L Chow, Kerry R Delaney, Michael J Hendzel, Zhaolan Zhou, Jeffrey L Neul, John B Vincent, Juan Ausió

**Affiliations:** Department of Biochemistry and Microbiology, University of Victoria, 3800 Finnerty Rd, Victoria, BC V8W 2Y2, Canada; Department of Biochemistry and Microbiology, University of Victoria, 3800 Finnerty Rd, Victoria, BC V8W 2Y2, Canada; Department of Biology, University of Victoria, 3800 Finnerty Rd, Victoria, BC V8W 2Y2, Canada; Departments of Oncology and Cell Biology, Faculty of Medicine and Dentistry, University of Alberta, 11560 University Ave, Edmonton, AB T6G 2H7, Canada; Molecular Neuropsychiatry & Development (MiND) Lab, Campbell Family Mental Health Research Institute, Centre for Addiction and Mental Health, 250 College St, Toronto, ON M5T 1R8, Canada; Vanderbilt Kennedy Center, Departments of Pediatrics, Pharmacology, and Special Education, Vanderbilt University Medical Center and Vanderbilt University, 1211 Medical Center Dr, Nashville, TN 37232, United States; Department of Biochemistry and Microbiology, University of Victoria, 3800 Finnerty Rd, Victoria, BC V8W 2Y2, Canada; Molecular Neuropsychiatry & Development (MiND) Lab, Campbell Family Mental Health Research Institute, Centre for Addiction and Mental Health, 250 College St, Toronto, ON M5T 1R8, Canada; Department of Biology, University of Victoria, 3800 Finnerty Rd, Victoria, BC V8W 2Y2, Canada; Department of Biology, University of Victoria, 3800 Finnerty Rd, Victoria, BC V8W 2Y2, Canada; Departments of Oncology and Cell Biology, Faculty of Medicine and Dentistry, University of Alberta, 11560 University Ave, Edmonton, AB T6G 2H7, Canada; Department of Genetics, Epigenetics Institute, University of Pennsylvania Perelman School of Medicine, 3400 Civic Center Blvd, Philadelphia, PA 19104, United States; Vanderbilt Kennedy Center, Departments of Pediatrics, Pharmacology, and Special Education, Vanderbilt University Medical Center and Vanderbilt University, 1211 Medical Center Dr, Nashville, TN 37232, United States; Molecular Neuropsychiatry & Development (MiND) Lab, Campbell Family Mental Health Research Institute, Centre for Addiction and Mental Health, 250 College St, Toronto, ON M5T 1R8, Canada; Institute of Medical Science, University of Toronto, 27 King's College Cir, Toronto, ON M5S 1A8, Canada; Department of Psychiatry, University of Toronto, 27 King College Cir, Toronto, ON M5T 1R8, Canada; Department of Biochemistry and Microbiology, University of Victoria, 3800 Finnerty Rd, Victoria, BC V8W 2Y2, Canada

**Keywords:** methyl CpG binding protein, MeCP2, Rett, PEST sequences, chromatin

## Abstract

Mutations in methyl-CpG binding protein 2 (MeCP2), such as the T158M, P152R, R294X, and R306C mutations, are responsible for most Rett syndrome (RTT) cases. These mutations often result in altered protein expression that appears to correlate with changes in the nuclear size; however, the molecular details of these observations are poorly understood. Using a C2C12 cellular system expressing human MeCP2-E1 isoform as well as mouse models expressing these mutations, we show that T158M and P152R result in a decrease in MeCP2 protein, whereas R306C has a milder variation, and R294X resulted in an overall 2.5 to 3 fold increase. We also explored the potential involvement of the MeCP2 PEST domains in the proteasome-mediated regulation of MeCP2. Finally, we used the R294X mutant to gain further insight into the controversial competition between MeCP2 and histone H1 in the chromatin context. Interestingly, in R294X, MeCP2 E1 and E2 isoforms were differently affected, where the E1 isoform contributes to much of the overall protein increase observed, while E2 decreases by half. The modes of MeCP2 regulation, thus, appear to be differently regulated in the two isoforms.

## Introduction

**Figure 1:**
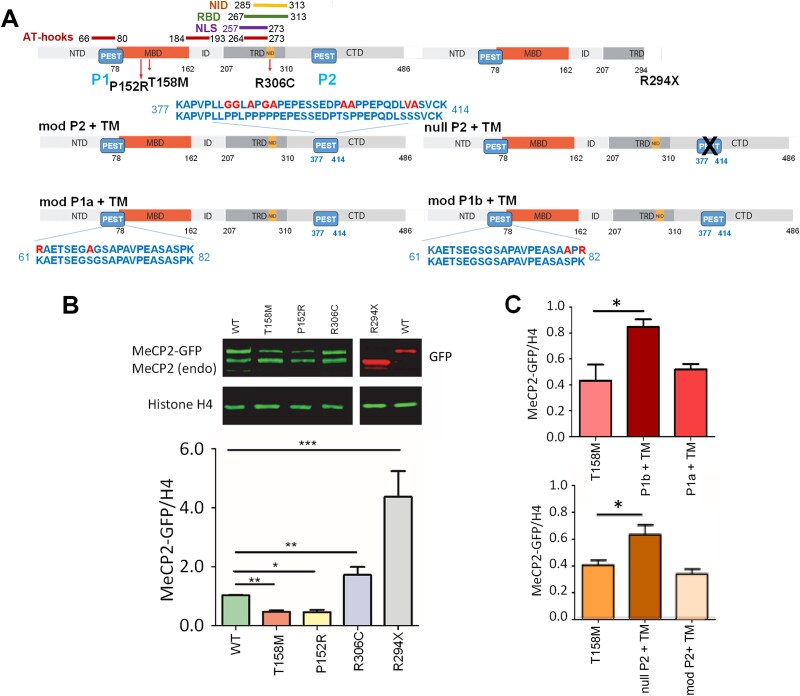
Comparison of MeCP2 expression levels in C2C12 cells transiently transfected with several GFP-tagged MeCP2 mutants. (A) Cartoon depicting the primary structure of MeCP2 and its constitutive NTD, MBD, ID, TRD, and CTD domains. The position of AT-hook, NID, NLS and the RNA binding (RBD) domains are indicated. A schematic representation of the different mutants and PEST-altered constructs used in this work is shown. Notice the absence of the entire NID and the CTD in the R294X mutant. (B) A representative western blot of isolated nuclei of C2C12 cells transfected with MeCP2 mutants is shown in (A). Below is a bar plot of the western blot analysis normalized by histone H4. MeCP2 antibody that binds to the Ct- epitope (green) and the GFP antibody (red) for R294X were used. (C) Bar plots, as in (B) for the MeCP2 PEST constructs, are shown in (A). Data are mean ± SEM; n = 5 biological replicates; one-way ANOVA with post hoc Dunnett's test; ^*^P ≤ 0.05, ^*^^*^P ≤ 0.01; ^*^^*^^*^P ≤ 0.001. For quantification of MeCP2, only the upper band, MeCP2-GFP, was used.

MeCP2 (Methyl-CpG binding protein 2) was identified based on its ability to bind to methylated DNA, in particular methylated CpG [1], but also to mCH (H = A, T or C) [2]. It is a vertebrate protein [3] with two isoforms, E1 and E2, that are the product of alternative splicing [4]. Despite its intrinsically disordered nature, it has been divided into six different domains (1, 3) (Fig. 1A). From the early studies, the protein was shown to interact with the transcriptional repressor Sin3a through its transcriptional repressor domain (TRD) [5], yet it also upregulates transcription by interacting with CREB1 transcriptional activator [6]. Mutations in MeCP2 account for most Rett syndrome phenotypes [7], and a crucial part of these mutations are clustered within the methyl-CpG binding domain (MBD) and TRD. Indeed, mice expressing a minimal version of the protein consisting only of the MBD and TRD show substantially improved neurological symptoms when introduced into a MeCP2-deficient mouse model of RTT [8]. Despite the well-established link between MeCP2 mutations and Rett syndrome (RTT), this fascinating protein has many other functions; a notable example is the classification of MeCP2 as a *bona fide* oncogene [9, 10] in several types of human cancer [11]. The protein also involves many other neurological disorders beyond RTT [12, 13]. Regardless of its ability to bind methylated DNA, MeCP2 is a global transcriptional regulator critical for proper brain function, in which the protein is predominantly found [14].

Most of the functional information on MeCP2 has come from early studies of its RTT-related mutations in knock-in mouse models [15, 16] and from mouse models where the expression of the protein was either abrogated entirely [17] or its MBD domain had deleterious mutations [18]. However, some essential enigmas remain within this context. For instance, in cortical neurons, MeCP2 is nearly stoichiometrically equivalent to core histones [19], and MeCP2 homeostasis has been shown to play a critical role in protein function [20]. Yet, how tightly a regulated balance is achieved remains unclear. Recent evidence indicates RTT mutations can jeopardize MeCP2 homeostasis [21, 22]. For instance, the low levels of MeCP2 observed in the brain of mice bearing the T158M missense variant, one of the most prevalent RTT mutations, has been shown to involve degradation by the proteasome system [21].

Hence, a potential mechanism partially involved in regulating MeCP2 homeostasis is degradation by the proteasome [23]. Proteins with relatively short half-lives, as is the case for MeCP2-E1 [24, 25], contain signal peptides known as PEST (Proline, Glutamic Acid, Serine, Threonine) sequences [26] that target them for degradation. Several years ago, we identified two PEST sequences in MeCP2 [27]. Interestingly, the two MeCP2 isoforms have different half-lives [25], and their degradation is likely differently regulated [24] by additional mechanisms. Regulation of MeCP2 homeostasis may be relatively complex, particularly concerning the E1 and E2 isoforms. A recent publication has described auto-regulatory feedback between the two isoforms [28].

In this study, we chose three RTT missense mutations, T158M, P152R, R306C and one nonsense mutation, R294X, which account for 30% of RTT cases [29]. By using an *in vitro* cell system and several mouse models for these mutations, together with some MeCP2 constructs with modified PEST sequences, we sought further insight into the potential involvement of these protein domains in the deleterious alterations of MeCP2 dosage [29].

## Results

### Different expression constructs of MeCP2 yield altered homeostasis *in vitro*

C2C12 cells provide an excellent *in vitro* model for the study of heterochromatin clustering, in particular for the involvement of methyl-CpG binding proteins [30]. MeCP2 is critical for chromatin organization in maturating cells and possibly stabilizes their differentiated state [14, 31], as is the case with differentiated C2C12 (myotubes) and neurons. We previously showed that this cellular system helps analyze alterations in MeCP2-chromatin binding for RTT missense mutations that fall within the MBD of MeCP2. Indeed, a correlation was established between the degree of structural binding impairment resulting from such mutations and their clinical severity [32].

Over the years, our lab has been interested in PEST sequences [26] that regulate MeCP2 degradation by the proteasome [27]. As mentioned, degradation by the proteasome has been observed in mouse brain cells carrying the T158M MeCP2 mutation [21]. Therefore, we wondered whether a similar effect could be reproduced in C2C12 cellular system and, if so, whether it could be used to test the PEST hypothesis [27]. The PEST hypothesis states: “MeCP2 turnover is regulated by proteolytic degradation through the 26S UPS and mediated via its two PEST domains” [27].

Our earlier work used the C2C12 cell line to study the molecular mechanistic and phenotypic correlation of missense RTT mutations [32]. In the present work, we look at the differing expression levels of MeCP2 mutants and study their correlation with the corresponding *in vivo* mouse models.

Figure 1A provides a schematic representation of the mutants and MeCP2-E2 constructs; because previous studies reported a significant reduction of MeCP2 levels associated with the T158M mutation [21], we began by quantifying MeCP2-E1 protein expression in C2C12 cells. As shown in Fig. 1B, the levels of T158M MeCP2 observed within this system (~40% compared to wild-type) recapitulate the levels present in the brains of the mutant mice (~40% at adulthood). This observation not only validates the use of this cellular system for our analyses but also indicates that the information about the levels of these mutants is not cell type-dependent; instead, it is encrypted by MeCP2. We therefore extended our approach to include other mutant MeCP2 constructs (P152R, R306C, and R294X; Fig. 1B) and to the MeCP2 constructs expressing different versions of altered PEST domains (Fig. 1C). As shown in Fig. 1B, the missense mutation P152R, which, like T158M, affects binding to methylated DNA, resulted in a similarly reduced MeCP2 expression. In contrast, the missense mutation R306C, which hinders the binding of the protein to the Nuclear Receptor Co-Repressor (NCoR) transcriptional repression complex, and the nonsense R294X mutation, which eliminates the TRD (Fig. 1A), exhibit enhanced levels of expression (~170% for R306C and ~4 fold increase for R294X).

For analyzing the PEST constructs, we used the T158M mutation expressing two differently modified PEST domains for the Nt- PEST (P1a, P1b) and a null and modified (null P2, mod P2) Ct- PEST (Fig. 1A). Whereas the P1b and null P2 both exhibit a substantial increase in protein levels relative to the control T158M, neither P1a nor mod P2 exhibit differences in their protein levels (Fig. 1C). Moreover, P1b almost completely recovered the level of expression of T158M but not to that of the wild type (WT) MeCP2. Yet, it is possible that the combined effect of the P1b and null P2 could fully rescue protein levels to near WT.

### DNA binding affinity of MeCP2 constructs

The increased accessibility of MeCP2 constructs to the proteasome described in the previous section suggests a decreased affinity for DNA. MeCP2 is an intrinsically disordered protein (IDP) that binds methylated DNA through the relatively small structurally organized MBD. The remaining disordered domains can acquire additional organization upon interaction with binding partners, including non-methylated DNA, RNA and protein cofactors [33], which are characteristics of IDPs [34]. Hence, mutations along the entire molecule can potentially result in structural modifications that reduce MeCP2's chromatin affinity, making the protein more accessible to the proteolytic machinery and susceptible to degradation [35].

**Figure 2:**
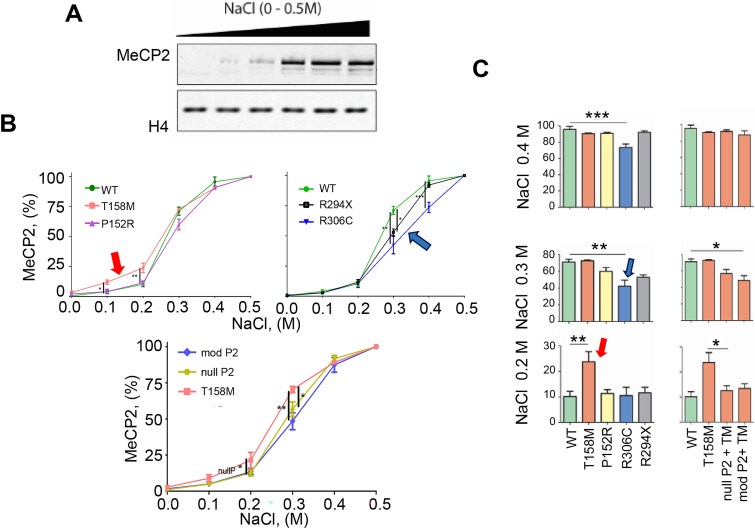
Ionic strength dependent DNA binding affinity of MeCP2 constructs within chromatin. (A) A representative western blot of the MeCP2 soluble fraction at different salt concentrations. (B) MeCP2 ionic-strength dependent elution patterns for the different mutants and constructs used in this work. (C) Bar plots of the data shown in (B) at three different NaCl concentrations for better data visualization and to emphasize the main differences exhibited by the different constructs. The nomenclature of the constructs is the same as in previous figures. One-way ANOVA with post hoc Dunnett’s test; n = 5 biological replicates; ^*^P ≤ 0.05, ^*^^*^P ≤ 0.01; ^*^^*^^*^P ≤ 0.001.

To analyze the DNA binding affinity of MeCP2 mutants and modified PEST constructs, we used differential salt extraction (Fig. 2) of C2C12 nuclei [36]. As shown in Fig. 2B and C, MeCP2 T158M has the lowest affinity, particularly at the lower ionic strengths (0.1–0.2 M NaCl, red arrows), consistent with previous findings in knock-in mice [15, 16]. However, R294X nonsense mutation and R306C missense mutation affecting the NID exhibit the highest binding affinity compared to wild-type MeCP2. The results of R294X agree with those reported in Fig. 1F of [22], demonstrating lower elution of R294X relative to wild-type.

We next assayed the DNA affinity of MeCP2 to DNA using constructs consisting of the impaired Ct- PEST domain (P2), which has the highest PEST score (+17.30 compared to +8.45 for the N-terminal PEST) [27] and contains the poly-proline II helical organization [37, 38], a key characteristic of these domains. Interestingly, both modified P2 constructs show a slight increase in the chromatin binding affinity of the MeCP2 T158M mutant (Fig. 2B, lower panel), suggesting how this domain indirectly enhances DNA binding. It remains to be experimentally determined whether the observed enhancement in DNA binding in the P2 PEST mutants is due to changes in the experimental isoelectric point (pI) of the protein since the interaction between MeCP2, a highly basic protein with pI of ~10 to DNA involves electrostatic interactions [39].

### MeCP2 clustering to chromocenters is impaired by MBD mutations and PEST alterations

**Figure 3:**
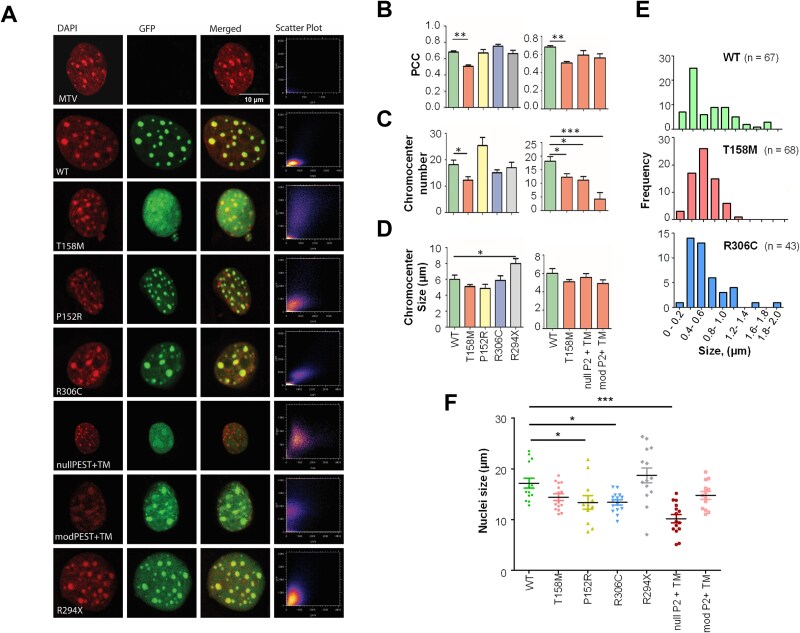
Confocal fluorescence microscopy of transiently transfected C2C12 with different MeCP2 constructs. (A) Confocal image stacks of DAPI (red), GFP (green), and merged channels. The scatter plot depicts the overlap of red and green pixels. MTV is the empty vector, pcDNA3.1-Ct-GFP. The missense mutations T158M and P152R are within the MDB, while R306C is part of the NID. The double mutants, modified PEST (mod P2) and null PEST (null P2), contain the missense mutation T158M. The nonsense mutation R294X is missing the critical NID and the entire CTD. The 10 μm scale length applies to all images. (B) Bar plot representations of Pearson correlation coefficient (PCC) of the DAPI and GFP within the nuclei for quantitative co-localization of MeCP2 to chromocenters. (C) and (D) are the bar graphs for the chromocenter size (~50 chromocenters measured) and chromocenter number of the nuclei, respectively. Data are mean ± SEM; n = 15–10 from three biological replicates. (E) Frequency of size distribution for three representative MeCP2 constructs: WT, T158M and R306C mutants. (F) A scatter plot of the different mutants' nuclear size (average diameter) distribution is shown in (A). Each point corresponds to an individual nucleus size (average diameter in μm). Data are mean +/− SEM, n = 11–15. One-way ANOVA with post-hoc Dunnett’s test. ^*^P ≤ 0.05, ^*^^*^P ≤ 0.01; ^*^^*^^*^P ≤ 0.001.

Similar to mature neurons [40], differentiated C2C12 cells are characterized by the presence of dense pericentromeric regions (chromocenters) [30]. Hence, they are useful for the study of MeCP2’s involvement in their formation [31]. Chromocenters can easily be visualized by their intense DAPI staining, making them amenable to co-localization analysis with GFP-tagged MeCP2 using fluorescence microscopy [32]. To this end, we used Pearson’s correlation coefficient (PCC) analysis [32] and the resulting scatter plots to assess co-localization quantitively (Fig. 3).

As previously reported, the T158M MBD mutation abolished clustering [16, 21, 32], whereas the P152R MBD mutation only impaired clustering. Mutations outside the MBD, R294X and R306C impaired chromocenter clustering. (Fig. 3A and E). The modified and null P2 PEST constructs exhibited an abolished clustering with a somewhat diffused MeCP2 distribution, which was more prominent for the null P2 PEST (Fig. 3A). The confocal fluorescence microscopy studies revealed variation of the nuclear size that was dependent on the mutation type of MeCP2 (Fig. 3F), albeit to a lesser extent in the tested RTT-causing mutations. The nuclei size of transfected cells mirrored their relative protein expression (Fig. 1B).

Our findings demonstrate that differentiated C2C12 cells are effective for studying MeCP2’s role in chromocenter formation, with mutations in the MBD and PEST domains significantly impacting chromocenter clustering and nuclear size, as evidenced by confocal fluorescence microscopy and Pearson's correlation coefficient analysis.

### FRAP analysis provides evidence for a rapid chromatin exchange of MeCP2 mutants with enhanced proteolysis

**Figure 4:**
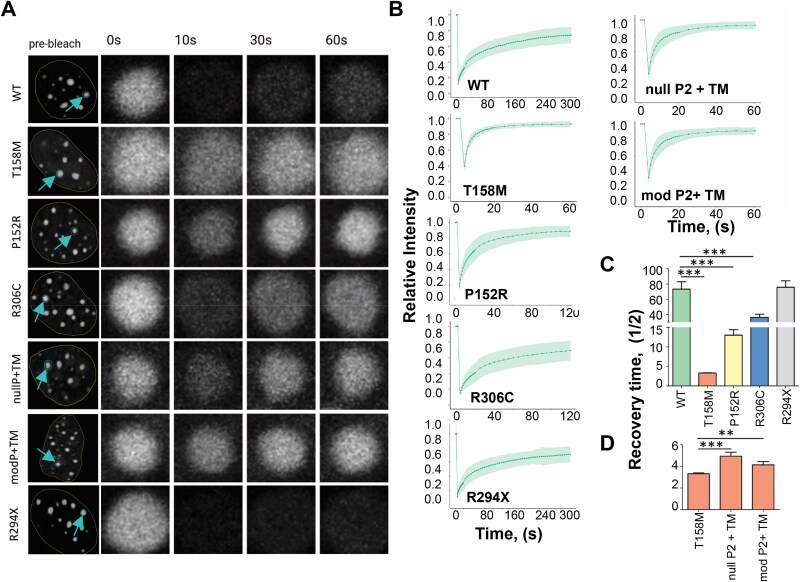
FRAP analysis of transfected C2C12 comparing the mobility and binding dynamics to chromocenters of different MeCP2 mutants and constructs. (A) Fluorescence recovery of the first 60 s of the GFP at the C-terminus of transfected MeCP2 after photobleaching. The blue arrow specifies the chromocenter that is targeted by the laser. The recovery for the first 60 s at 0 s, 10 s, 30 s, and 60 s is shown next to the pre-bleached full view of the nucleus. (B) FRAP recovery curves of MeCP2 constructs at the chromocenters; n = 30–32. (C). Bar graph comparisons of half recovery time of GFP in MeCP2 constructs. One-way ANOVA with post hoc Dunnett’s test. Comparisons were made only to WT and T158M. (D) Same as (C) except comparisons are made to T158M as both double mutants, nullPEST and modified PEST, contain the missense mutation T158M. Unpaired t-test. P-values for nullPEST and mPEST are < 0.0001 and 0.0045, respectively. Data are mean ± SEM; ^*^^*^P ≤ 0.01 ^*^^*^^*^P ≤ 0.001.

In the previous sections, we addressed the amount of protein expressed, the binding affinity and the cellular distribution of the constructs, which could only hint at their potential dynamics inside the nucleus. The FRAP analysis for MeCP2 constructs, shown in Fig. 4A, provides an alternative method to better understand protein dynamics by measuring the mobility of the MeCP2-GFP constructs within the nucleus.

Figure 4A shows the nuclei sample analyzed, and Fig. 4B shows FRAP recovery curves for MeCP2 constructs at the chromocenters (indicated by an arrow in Fig. 4A). As seen in Fig. 4C and D, the half-time of recovery of the wild type (73 ± 10 s) and R294X (76 ± 9 s) are indistinguishable (mean ± SEM). In contrast, all the other missense mutations have lower recovery times, with R306C (36 ± 4 s), P152R (13 ± 1.5 s), and T158M at (3.3 ± 1 s). When comparing the half-recovery time of the double mutants nullPEST P2 and modPEST P2 to T158M, the difference is within 2–3 s (Fig. 4D). The increased recovery time suggests that the Ct-PEST (P2) domain plays a role in stabilizing DNA binding. Notably, the bleaching for all mutants with the T158M mutation (T158M, mod P2 and nullP2) was not as robust as other constructs. The relative intensity curve shows the fluorescence intensity is ~0.4 following bleaching, whereas for other mutants and wild-type, the intensity is ~0.2. The higher relative intensity suggests high mobility of MeCP2 when it has the common Rett-causing mutation, T158M.

Overall, the FRAP analysis of MeCP2 constructs provides insight into the binding dynamics and diffusion of MeCP2 to the densely methylated pericentromeric regions. Mutations that impair DNA binding, such as those within the MBD, allow MeCP2 to quickly diffuse and, therefore, allow for a faster recovery than other mutations that do not affect binding of MeCP2 to DNA, such as the R294X and R306C.

### A complementary view from the in vivo mouse models

**Figure 5:**
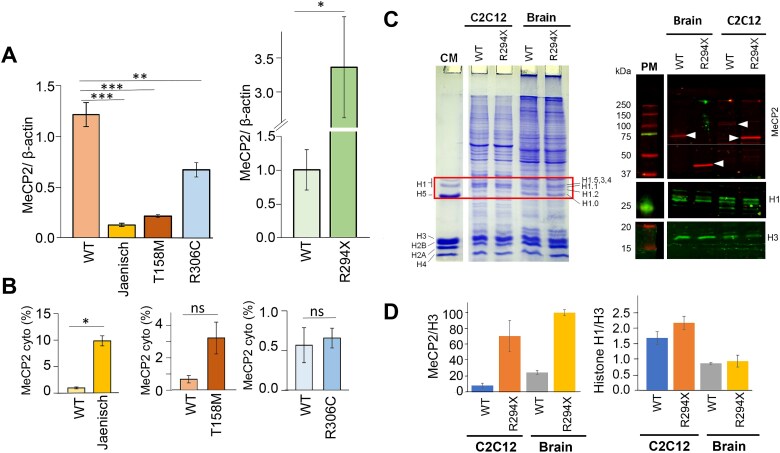
Comparison of MeCP2 levels using *in-vivo* mice model*.* (A) Mean values of the MeCP2/β-actin levels observed in the brains of the Jaenisch (*Mecp2*^tm1.1Jae/y^), T158M (*Mecp2*^T158M/y^), R306C (*Mecp2*^R306C/y^) and R294X (*Mecp2*^R294X/y^) MeCP2 mutant mice respectively, compared to the brains of the original *Mecp2*^+/y^ mice strains used to generate the mutant mice. (B) Percentile of cytoplasmic MeCP2 was determined as in [41]. No cytoplasmic MeCP2 was detected in R294X. (C, left) SDS-PAGE of the nuclear proteins from C2C12 cells transfected with a MeCP2 R294X plasmid and (*Mecp2*^R294X/y^) brain compared to their wild-type counterparts. CM, chicken erythrocyte histone marker. (C-right) Western blot of the gel shown on the left side, using N-terminal MeCP2, histone H1 cocktail and histone H3 antibodies. PM, protein marker (the molecular weights in kDa of the different bands are indicated). (D) Bar plots of the MeCP2/Histone H3 and histone H1/histone H3 ratios. The error bars correspond to the standard deviation obtained with two sample replicates.

We decided finally to extend our analyses by using *in vivo* transgenic P40 male mouse whole brains, with the *MeCP2* T158M and R306C mutations as analyzed in the previous sections. We included another P40 male mouse model (*Mecp2*^tm1.1Jae/y^) [18] in which exon 3 of *MeCP2*, encoding 116 amino acids encompassing much of the structured MBD, was deleted (referred as the Jaenisch model). Hence, it provides an excellent system for studying the impairment of MeCP2-DNA binding in the chromatin context [41]. Lastly, whole brain of male P30 mouse with R294X MeCP2 were also analyzed for total MeCP2 expression. As in Fig. 1, we first looked at the protein expression levels (Fig. 5A). Here, MeCP2 from all three mutant strains showed significantly diminished levels compared to WT (in the C2C12 system transfected R306C showed increased levels, Fig. 1B). We also looked at the cytoplasmic compartmentalization of MeCP2 compared to wild type for each of these mutants [41]. In agreement with our earlier report, we observed increased (~10%) MeCP2 in the cytoplasm of the brain cells of the Jaenisch mouse model. A more modest increase (~3%) was observed for MeCP2 T158M, but for R306C and R294X, the amount of MeCP2 was indistinguishable from wild-type strains used to generate the mutant mice. The T158M/y and Jaenisch mouse models showed residual protein in the cytoplasm [see (8) and (Fig. 5B)] relative to R294X and R306C, which is in agreement with previous findings that the translocation of MeCP2 into the nucleus does not depend on its NLS but rather on an intact (and correctly folded) MBD [42].

Because of the significant expression of MeCP2 R294X in both C2C12 (Fig. 1B) and mouse brain (Fig. 5A), we decided to explore whether such dramatic protein increase would result in the displacement of histone H1, present in the cells from both systems. This experiment was intriguing given the similarity of binding of both chromosomal proteins, MeCP2 and H1, to their chromatin binding sites [43] and a proposed competition between them [43, 44]. The results are shown in Fig. 5C and D. The bar plots (Fig. 5D) clearly show that the amount of histone H1 remained constant in the nuclei from both the C2C12 cells transfected with the R294X expression vector or in the cells from the (*Mecp2*^R294X/y^) mouse brain.

### The effect of MeCP2 mutations on the E1 and E2 isoforms

**Figure 6:**
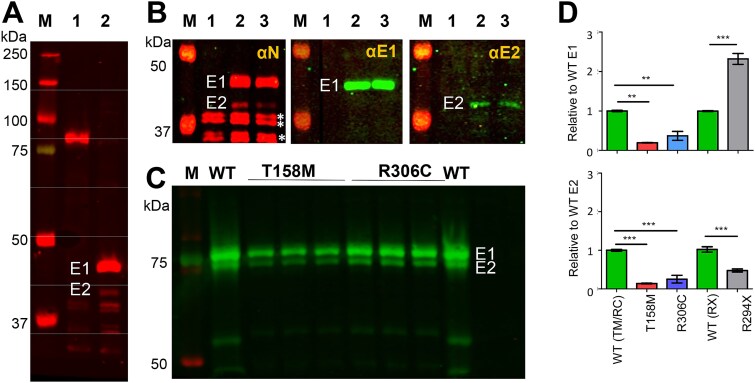
MeCP2-E1/-E2 ratio in mice mutants. (A) Western blot, using an antibody against the N-terminus of MeCP2, of male mice brain expressing the R294X mutation. Lane 1 *Mecp2*^+/y^ and lane 2 *Mecp2*^R294X/y^. M, protein marker. (B) Detail of similar westerns as in (A) stained with the N-terminal antibody (αN), MeCP2-E1 (αE1) and MeCP2-E2 (αE2) specific antibodies. Lane 1: *Mecp2*^+/y^ lanes 2–3: Two samples of *Mecp2*^R294X/y^ and M, protein marker. (C) Western blot using generic MeCP2 antibody (Sigma-Aldrich) for the wild-type used to generate the *Mecp2*^T158M/y^ and *Mecp2*^R306C/y^ and an n = 3 (different brains) for each is shown. (D) Bar plot representation of the MeCP2-E1 and MeCP2-E2 relative to WT in the mouse Rett models brain of the mutants. WT (TM/RC) is the same wild-type as in (C). WT (RX) is the wild-type strain used to generate *Mecp2*^R294X/y^.

Finally, taking advantage of the detectable expression of the MeCP2 E1 and E2 isoforms in the mouse models, we resolved the electrophoretic bands for each isoform by running for an extended period in 12% SDS-PAGE gels (see Experimental Procedures) (Fig. 6). The two bands were identified using isoform-specific antibodies prepared in house [25] (Fig. 6B). As the bar plots in fig. 6D indicate, both MeCP2-E1 and MeCP2-E2 decrease in the T158M and R306C while in the R294X mutant it appears that the overall MeCP2 increase observed (Fig. 5A) can be mainly accounted by an increase in the E1 isoform and the E2 isoform decrease by 50% when compared to the amounts present in the WT.

## Discussion

### MeCP2 mutations affect cellular protein levels

MeCP2 homeostasis is a critical parameter for proper MeCP2 function, and although it can oscillate within a limited range [45], large deviations can be detrimental. Although the range of normal variation tolerance is not well established, the results from the analysis of MeCP2 circadian cycle-dependent variation in mouse brains suggest such variation to be approximately ±30% in mice at any given time [46]. In the present study, the variation for all the mutations studied (except R306C in C2C12 cells) is far beyond those limits (Figs 1B and 5A).

Despite both P152R and T158M MeCP2 being present in C2C12 cells at similarly decreased levels (Fig. 1B), these mutants exhibit different chromatin binding and affinity characteristics (Figs 2 and 4). The low binding affinity of the T158M (in particular below 0.25 M NaCl; Fig. 2B and C), likely reflects the binding of the molecule to non-methylated DNA regions [47, 48], as MeCP2 binds to non-methylated DNA with approximately a ~ 3 fold lower affinity than to methylated DNA [47, 48]. In addition, in contrast to P152R, the T158M molecular transition is highly destabilizing to the MBD, as T158 occupies a critical position that coordinates two consecutive turns of an Asx-ST turn motif [49, 50], resulting in disrupted binding to methylated DNA [50]. This observation agrees with its more diffuse nuclear distribution and defective chromatin clustering (Fig. 3A), as described earlier [32]. The more modest effect on the binding affinity of P152R is also reflected in its higher FRAP ½ recovery time (13 s) when compared to T158M (3.2 s) (Fig. 4D), which could account for the higher binding affinity of this MeCP2 MBD mutant for methylated DNA compared to T158M [51]. Nevertheless, the overall striking effects of these two MBD missense mutations on the ½ time of chromatin residency (Fig. 4) make these proteins more amenable to proteasome degradation and underscore the role of degradation machinery in their low cellular levels (Figs 1B and 5A) with functionally detrimental consequences [21]. The results for the two MBD missense mutations analyzed here agree with those obtained for a recently described MeCP2 G118E RTT mutation. When expressed in a knock-in mouse line, reduced MeCP2 levels and partially impaired DNA binding were observed [52]. All these findings emphasize the relevance of MeCP2’s MBD to DNA binding compared to other DNA/RNA binding domains of the protein. In addition to its MBD, MeCP2 contains three AT-hook-like domains [53] and an RNA binding domain [54–56] outside of the MBD (Fig. 1A).

The results obtained with the expression of MeCP2 R306C in C2C12 (Fig. 1B) agree with the overexpression observed in knock-in mice expressing MeCP2 R306C-GFP [57]. However, they disagree with the decrease we observed in Fig. 5A, which reflects the results in [58] using an R306C mouse model and where no overall change was shown, similarly observed in Vecsler *et al.* [59]. From these results, it appears that the presence of C-terminal GFP might be responsible for these discrepancies in protein levels, which would be unsurprising given the proximity of the bulky GFP to the mutation site at R306. Nevertheless, the mutant protein binds more tightly to DNA (Fig. 2B and C) than any other mutants or the wild-type, and it shows a very well-defined co-localization at chromocentres (Fig. 3A and B) despite its highly dynamic chromatin binding (t^1/2^ = 33.9 s).

The high presence of R294X MeCP2 is equally intriguing (Figs 1A and 5B). However, from the perspective of consistency between the cell and mouse model, they did not appear affected by the presence of C-terminal GFP, as both models exhibited higher protein levels relative to wild-type.

### The PEST sequences as a rheostat of MeCP2 degradation

We have recently reviewed in detail the involvement of MeCP2 ubiquitination and sumoylation in this protein homeostasis, particularly as it pertains to the PEST domains [38].

The alterations in expression for the two P1 and P2 PEST domain constructs (Fig. 1A) are interesting. In the case of P1, mutation of S80 and K82 to alanine and arginine, respectively, prevented the (S/T) phosphorylation signalling (at S80) required for ubiquitination of the adjacent lysine (K82), which is necessary for recognition for degradation by the proteasome [26]. There is experimental evidence for S80 phosphorylation [60] and K82 ubiquitination [61]. Therefore, it makes sense that in their absence, the protein amounts increases (Fig. 1C).

However, the guess it was made in our review this would be similar for two potentially equivalent sites (K61 and S68) as prerequisites of PEST-mediated degradation (Fig. 1A) [38] proved to be negative, as MeCP2 abundance remained the same. For the P2 constructs, only null P2 resulted in a significant protein increase, as expected (Fig. 1C), but alteration of the polyproline residues had no effect. Moreover, the increase in the binding affinity of these two constructs (Fig. 2B) supports the molecular mechanism described for PEST domains [62]. Accordingly, the primary structure around the PEST region (polyproline) is finely tunable by phosphorylation that results in a loss of conformational stability, which is what ultimately modulates the degradation process by the proteasome rather than recognition of the phosphate PTM [62]. Finally, the higher expression of R294X mutant where the PEST P2 domain is absent also supports the role of this domain in regulating internal nuclear homeostasis.

In conclusion, the results described in this work provide initial support for the involvement of the PEST sequences in MeCP2 degradation [27] in contributing to the protein homeostasis. However, further work in this direction is still needed. In this regard, it would be of interest to look at the phosphorylation and ubiquitination turnover using the PEST mutant MeCP2 in transfected cell lines as described here in conjunction with mass spectrometry to detect the PTM profile of mutated MeCP2.

### The RTT MeCP2 mutations affect nuclear size

An interesting observation of the fluorescence work was the nuclear size dependence as a function of the MeCP2 RTT mutation constructs expressed in C2C12 (Fig. 3F). The mirroring trend of the nuclear size (Fig. 3) to the amount of expressed protein (Fig. 1B) suggests an essential role of MeCP2 on nuclear size [63]. Indeed, neurons expressing an MBD truncated version of MeCP2 are deficient in the expression of this truncated MeCP2 (Fig. 5A) and exhibit a significantly reduced nuclear size of about 20% [41]. This correlation between nuclear size and protein level is in contrast to what is observed with the null PEST P2 that while it resulted in a statistically significant protein level increase (63 ± 25%) relative to T158M (Fig. 1*C*), the nuclear size decreased (30 ± 5%). This discrepancy can be explained by the diffuse nuclear distribution of the mutated protein (Fig. 3A), which, together with its higher recovery time (Fig. 4D), indicates its dynamically loose presence within the nucleus with a lower chromatin architectural organization.

It has been described that some factors that structurally organize and modify chromatin might also influence nuclear size and morphology [63]; an extreme example of which can be provided by protamines during the nuclear condensation undergone during spermiogenesis [64].

Thus, in addition to its many functions, MeCP2 appears to play an essential architectural role wherein its effects on chromatin architecture [65] and that on the higher order genome [66] indirectly impact the overall nuclear size.

### The amount of nuclear histone H1 is not affected in cells over-expressing MeCP2 R294X, providing further support for MeCP2/histone H1 competition hypothesis

Neurons, particularly cortical neurons, contain the largest amount of MeCP2, which is experimentally estimated to be 16 × 10^6^ molecules per nucleus, consisting of 32 × 10^6^ nucleosomes and 40 × 10^6^ methylated CpG sites [19]. Such values would correspond to approximately one MeCP2 molecule per two nucleosomes. Following these calculations, the value of MeCP2 R294X overexpression (~4-fold) considering the average of MeCP2/H4 observed in C2C12 transfected cells at confluence (~4.3) (Fig. 1B) and in the mouse *Mecp2*^R294X/y^ (~3.4) (Fig. 5B), would approximately correspond to two molecules of MeCP2 R294X per nucleosome. This 2:1 ratio of MeCP2 to nucleosome means that chromatin is saturated with this protein in the nucleus of the cells expressing this MeCP2 mutant, where it does not appear to replace/displace histone H1 (Fig. 5D and E). This similar amount of H1 in R294X compared to wild-type MeCP2 is a critical observation of the controversial competition between these two proteins in the *in vivo* setting.

A potential competition between MeCP2 and histone H1 was postulated by Nan *et al*. in 1997 [44] using two methylated and un-methylated plasmids chromatinized by incubation in a *Xenopus* oocyte extract, and the reconstituted complexes were then used in competition experiments between these two proteins. It was concluded that in these constructs, MeCP2 could displace histone H1 in a DNA methylation-dependent way [44]. Of note, the average nucleosome repeat length (NRL) of the reconstituted chromatin complexes used in this work was determined to be 160 bp, which questions the validity of the observations, as histone H1 cannot form chromatosomes (unable to bind to its proper site in native chromatin) on an NRL smaller than 167–168 bp [67]; hence its binding to 160 bp-long constructs is likely non-specific. Subsequently, it was observed that neurons from a *Mecp2* knock-out mouse contained approximately double the amount of histone H1 compared to wild-type mice. Cortical neurons had been previously shown to contain approximately half the amount of histone H1 present in most other somatic cells [68, 69]. The doubling of the histone H1 in neurons of *Mecp2* KO mouse was considered to support the competition proposal [19, 43]. However, MeCP2 increases gradually during neuronal development and the transition that brings chromatin to half of its histone H1 content is very sharp, occurring within a very short period at birth [70]. Following the competition thread, Ghosh *et al.* carried out fluorescence anisotropy on *in vitro* reconstituted oligonuclesomes and purified MeCP2 and histone H1.0, as well as FRAP-kinetics *in vivo* analyses of cells microinjected with differently labelled MeCP2 and histone H1.0 [43]. They first indicated that a nucleosome occupied by H1.0 cannot be co-occupied by MeCP2 and vice versa, whereas FRAP was consistent with a competitive exclusion of MeCP2 by H1.0. However, these findings are difficult to explain because although the dynamic of chromatin binding for linker histones H1.0, H1.4 and H1.5 of 65–80 s is similar to that of wild-type MeCP2 (Fig. 4C) [71–73], the midpoint for the salt elution of histone H1 from chromatin is 0.45–0.5 M NaCl with a sharp elution profile [36, 74]. Moreover, such an elution pattern is quite different from the shallower one of MeCP2 with a midpoint elution of 0.3–0.4 M NaCl depending on the cellular type [36] (see also Fig. 2B).

More recently, to unequivocally test the MeCP2-histone H1 competition hypothesis *in vivo*, chromatin immunoprecipitation of forebrain excitatory neurons followed by sequencing using a Flag-tagged H1.0 in transgenic mouse was performed [75]. The results using this system showed clearly that Flag-H1.0 and MeCP2 occupied similar genomic regions and that the functional binding of MeCP2 and H1.0 are largely independent [75], in agreement with the co-localization of MeCP2 and histone H1 that we recently observed in a ReNCell system [76] and as shown in the current study Fig. 5C and D. Moreover, Flag-H1.0 binding was unchanged upon MeCP2 depletion, and mild overexpression of the former did not affect MeCP2 binding [75].

The estimated number of two MeCP2 molecules per nucleosome observed in the Mecp2R294X/y mice likely represents an upper limit of co-occupancy of MeCP2 and histone H1 in chromatin. This number might be accommodated by the smaller 294 amino acids of the mutant protein compared to the 498 amino acids of the WT protein and the absence of the P2 PEST (Fig. 1A). Yet, as to how this level is achieved in C2C12 cells transfected with R294X MeCP2 and in the brain of *Mecp2*^R294X/y^ mice, remains to be experimentally elucidated.

### MeCP2 mutations affect its isoform ratios

An intriguing observation in this study concerns the effect of the mutations on MeCP2 isoforms. Our ability to distinguish between these two isoforms, which differ only in their 21 (MeCP2-E1) and 9 (MeCP2-E2) Nt- amino acids, using western blotting (Fig. 6A–D), suggests that these short regions exhibit a different secondary structure as these isoforms are affected differently by the mutations.

Our ability to distinguish between the two MeCP2 isoforms gave us a unique opportunity to examine how RTT-causing MeCP2 mutations affect these two isoforms.

In the T158M and R306C missense mutants, both MeCP2 E1 and E2 isoforms appear to be similarly affected (Fig. 6D), suggesting that they are subject to proteolysis to a similar extent, as would be expected if their degradation by the proteasome is modulated by the two PEST regions which are present in both. This is quite different from what is observed in the R294X mouse model (Fig. 6D), where an overall MeCP2 increase is observed in this instance (Figs 1B and 5C), which appears to be the result of the E1 isoform overexpression while E2 decreases by half suggesting that in this mutant E2 is less affected by the ablation of the PEST P2 sequence.

Given the similarity between the results obtained here by expression of the different mutants in C2C12 cells and the mouse models, it would be interesting to express mutants using the E2 form rather than E1 to study the differential role played by their varying N-terminal regions in all the processes explored here.

## Material and methods

### Cell culture and transfection

Mouse myoblasts, C2C12, were grown at 5% CO_2_ at 37°C in DMEM high glucose (Invitrogen; 11965092) with 10% FBS (Sigma-Aldrich; F1051). Cells were 50%–70% confluent at the time of transfection with lipofectamine 3000™ (Invitrogen; L3000015) which was carried out according to the manufacturer's protocol. Human MeCP2-E1 constructs expressing the mutations: T158M, P152R, R306C, modP2 + TM, nullP2 + TM, modP1a + TM, mod P1b + TM and the nonsense mutation R294X (see Fig. 1A) were cloned onto expression vector pcDNA3.1_Ct-GFP_AP717. Cells were harvested 48–52 h after transfection, and they were subsequently analyzed by confocal microscopy or used in other biochemical analyses. The possibility of the GFP interference with protein binding and dynamics is minimal as both the *in vivo* or *in vitro* analysis of the tagged protein did not show significant differences from MeCP2 with no tags [32, 77].

### Mice

P30 male mice, strain C57BL/6, containing the MeCP2 R294X mutation (*Mecp2*^R294X/y^) were obtained as described in [22]. Animal care procedures for *Mecp2*^R294X/y^ mice were approved by the Van-derbilt Animal Care and Use Committee. Mice were housed in AAALAC-approved facilities at Vanderbilt University. P40 male mice, strain C57BL/6, consisting of the T158M and R306C mutations (*Mecp2*^T158M/y^ and *Mecp2*^R306C/y^) were generated as described in [21] and in [16, 58], respectively. Experiments for *Mecp2*^T158M/y^ and *Mecp2*^R306C/y^ mice were conducted following the ethical guidelines of the NIH and with the approval of the IACUC of the University of Pennsylvania. P40 male mice with mixed genetic backgrounds (C57BL/6 and BALB/c) that had an abrogated MBD domain (*Mecp2*^tm1.1Jae/y^) [18] were obtained from mutant mouse resource and research center at the University of California, Davis. (MMRRC, UC Davis) [41]. Experimental protocols for *Mecp2*^tm1.1Jae/y^ mice were approved by the Animal Care Committee at the University of Victoria. All experiments were performed per guidelines from the Canadian Council of Animal Care.

### Confocal fluorescence microscopy

Cells were fixed on coverslips (Bellco Glass Inc; 501944702) in 4% paraformaldehyde (Electron Microscopy Sciences; 15170) in PBS at −20°C for 10 min. Following three washes with PBS, cells were stained with DAPI. (Invitrogen; D1306) to a final 1 μg/ml concentration. The cell membrane was lysed with triton-X to minimize background auto-fluorescence to a final concentration of 0.1% triton in PBS. Cells were incubated with the diluted DAPI and triton mixture for 20 min at room temperature in the dark. Cells were washed with PBS three times, and coverslips were mounted face down on a glass slide using Immu-Mount™ (Thermo Shandon Limited; 9990402) and were stored in a dark place at 4°C until imaging. Confocal fluorescence images were taken using CFI Plan Flour 100XS Oil (NA 1.3) Nikon objective lens on the Nikon C2 confocal microscope. Channel 1 (DAPI) had a laser wavelength of 405 nm and laser power of 10, while channel 2 (GFP) had a laser wavelength of 488 nm and a laser power of 5. Images were taken in Z-stacks with a 2 μm step size. Pixel width and height were 0.103 μm, and the image size was 2048 pixels. Images were captured with NIS-Elements imaging software (https://www.microscope.healthcare.nikon.com/products/software/nis-elements). The scatterplot for measuring the nuclear size determined from the average diameter of the transfected C2C12 was determined from measurements done by ImageJ from the DAPI channel of the transfected cells.

### Pearson correlation coefficient and scatter plots

Co-localization of MeCP2 to DAPI stained chromocenters was analyzed with ImageJ (https://imagej.nih.gov/ij/download.html). The background was subtracted by measuring its mean and removing it from the image. The Co-localization Finder plugin generated the scatter plot and Pearson correlation coefficient (PCC).

### FRAP analysis

For the FRAP analysis of wild-type and mutant MeCP2 proteins, C2C12 cells were seeded and transfected at approximately 60%–70% confluency on 35 mm MatTek dishes. The Qiagen Effectene transfection kit was used with some modifications to the protocol. 100 𝜇l of EC buffer was added along with 3 𝜇l of Enhancer and 1 𝜇g of DNA. This was incubated at room temperature for 20 min. 5 𝜇l of Effectene was added, and the solution was incubated for another 20 min at room temperature before being added to the cells. Media was changed the following morning, and FRAP experiments were performed using a Zeiss LSM710 laser scanning confocal microscope with a 63 × 1.4 NA Oil DIC Plan-Apochromat objective lens and a fluorescent photon multiplier tube detector. Entire heterochromatin domains were bleached with a 488 Argon laser. 3 pre-bleach images were collected, and then time-lapse images were collected every second for the first 20 s and then every 5 s for the remainder of the FRAP experiment. During imaging, cells were maintained in a live cell chamber at 37°C with 5% CO_2_. FRAP curves were normalized to the intensity of the whole nucleus throughout the FRAP experiment to account for the FRAP ROI and photobleaching of the entire cell as the result of repeated imaging.

### Nuclei isolation

Whole mouse brains previously frozen at −80°C, or fresh nuclei of C2C12 cells were homogenized with a Dounce in 4 volumes of lysis buffer A (0.25 M sucrose, 60 KCl, 15 mM NaCl, 10 mM MES (pH 6.5), 5 mM MgCl_2_, 1 mM CaCl_2_, 0.5% triton X-100) containing protease inhibitor cocktail at 1:100 [Complete™, EDTA-free Protease Inhibitor Cocktail, Roche; 5056489001], and a phosphatase inhibitor at 1:50 (PhosSTOP ™ Roche; 4906837001), and incubated on ice for 3–6 min. Lysates were centrifuged at 600 × g for 5 min at 4°C. Additional steps were carried out as in [41]. Nuclear DNA concentrations were determined by diluting the nuclei with distilled water. After a brief vortexing, SDS was added to a final concentration of 0.2% and the absorbance at 260 nm was measured on CARY 1 BIO UV–VIS to estimate nuclei concentration using a DNA extinction coefficient of A_260_ of DNA = 20 cm^2^mg^−1^ [78].

### Alkaline phosphatase (AP) treatment

Nuclei were isolated as described above, with the exclusion of phosphatase inhibitor from buffers A and B. Isolated nuclei were then treated with alkaline phosphatase (Antarctic Phosphatase, New England Biolabs Inc; M0289S) at a concentration of 1 U/5 ug nuclei. AP treatment was performed following the manufacturer's protocol.

### NaCl extraction of MeCP2

60 μg of isolated nuclei were diluted in buffer B with protease inhibitor and aliquoted in 6 fractions. Equal volumes of 2× NaCl solutions ranging from 0.2 M to 1 M in 20 mM Tris–HCl (pH 7.5), 0.5 mM EDTA, 2 mM DTT, and protease inhibitor were added to fractions. Fractions intermittently vortexed for 10 s every 5 min for three rounds and stored on ice. Samples were then incubated on ice for 30 min and centrifuged at 16 000 × g for 15 min at 4°C. The supernatant was separated and mixed with 2× sample buffer [250 mM Tris- HCl (pH 6.8), 4% SDS, 40% glycerol, 2.86 M β-mercaptoethanol, and 0.4% bromophenol blue]. The pellet of each fraction was resuspended in 1× sample buffer (125 mM Tris–HCl pH 6.8, 2% SDS, 20% glycerol, 1.43 M β-mercaptoethanol, and 0.2% bromophenol blue), sonicated for 2 min. All samples thus prepared were incubated at 100°C for 3 min before loading onto SDS gel.

### HCl extraction of nuclear proteins

Nuclei in buffer B were centrifuged at 600 × g. The pellet was homogenized with 0.6 N HCl using a Dounce. The sample was centrifuged at 7850 × g for 10 min at 4°C. The proteins in the supernatant were precipitated by adding of 6 volumes of acetone and overnight incubation at −20°C. Next day the precipitate was centrifuged at 16 000 × g for 10 min at 4°C and the pellet was resuspended in another six volumes of fresh acetone (to remove any residual HCl) and centrifuged at 16 000 × g at 4°C for 10 min. The pellet thus obtained was speed vacuum-dried with a Jouan RC 1010 speed-vac for 15 min at room temperature and stored at −80°C.

### SDS-page

Samples were analyzed by SDS-PAGE [acrylamide 15% separating, 4% stacking (30:0.8 acrylamide bis-acrylamide)] according to [79]. Gels were run at room temperature in (0.38 M glycine, 0.05 M Tris-base, 0.1% SDS) for 2 h at 100 V. Gels were stained with Coomassie Blue stain (25% isopropanol, 10%, 10% acetic acid, 0.27% w/v Coomassie Brilliant Blue G-250)) for at least 1 h and de-stained with a solution containing 10% isopropanol and 10% acetic acid for 1–3 h. For the separation of the MeCP2-E1 and -E2 isoforms at high resolution, 12% acrylamide separating gels were used and were run for 7 h (4.5 h for the R294X mutant) at room temperature and 115 V.

### Western blotting

Proteins were transferred onto a nitrocellulose membrane with Tris-glycine transfer buffer (40 mM Tris, 192 mM glycine, 20% methanol) for 2 h at 400 mA or overnight (16 h) at 100 mA. The membrane was blocked with 5% skim milk in PBST (137 mM NaCl, 2.7 mM KCl, 10 mM Na_2_HPO4, 1.8 mM KH_2_PO_4_, 0.1% (w/v) Tween). The primary antibodies were incubated overnight at 4°C and include anti-MeCP2 (Sigma Aldrich; M9317: 1:8000 dilution); N-terminal MeCP2 antibody MECP2 Monoclonal Antibody [(4B6) (H00004204-M01) 1:2000 Thermo Fisher Scientific], H4 (in-house; 1:50000); GFP clone 4B10 (Cell Signalling; 2955S; 1:1000 dilution). For the detection of histone H1, we used a cocktail consisting of a mixture of histone H1 antibodies: H1.1, H1.3 and H1.5 (kindly provided to us by Albert Jordan); H1.2 (PA5- 32009, Thermo Fisher Scientific) and H1.4 (PA5-44881Thermo Fisher Scientific). The mixture was used at a 1:2000 dilution. Secondary antibodies (IRDye 800 Anti-rabbit; Licor; 611-132-122; 1:10000 dilution/IRDye 680 anti-mouse; 926-68070; 1:5000/ECL Anti-Rabbit IgG, Horseradish peroxidase; GE Healthcare; NA934V; 1:2000) incubation was at room temperature for 1 h with shaking. Images were analyzed with Li-Cor Odyssey (LI-COR Biosciences Lincoln, NE, USA) and Li-Cor Image Studio Lite 5.2.5 software.

### Statistical analysis

Results were expressed as mean ± s.e.m as indicated with α < 0.05. GraphPad Prism software performed one-way analysis of variation (ANOVA) with post hoc Dunnett’s test, which compares values to the wild-type, and post hoc Tukey’s test, which compares all values (https:/www.graphpad.com). Statistical power calculation was completed on the R-studio “pwr” plugin to calculate the number of observations needed for a power of 0.8.
