# Correction to: Testing the PEST hypothesis using relevant Rett mutations in MeCP2 E1 and E2 isoforms

**DOI:** 10.1093/hmg/ddaf072

**Published:** 2025-05-05

This is a correction to: Ladan Kalani, Bo-Hyun Kim, Alberto Ruiz de Chavez, Anastasia Roemer, Anna Mikhailov, Jonathan K Merritt, Katrina V Good, Robert L Chow, Kerry R Delaney, Michael J Hendzel, Zhaolan Zhou, Jeffrey L Neul, John B Vincent, Juan Ausió, Testing the PEST hypothesis using relevant Rett mutations in MeCP2 E1 and E2 isoforms, *Human Molecular Genetics*, Volume 33, Issue 21, 1 November 2024, Pages 1833–1845, https://doi.org/10.1093/hmg/ddae119

The following changes have been made to the originally published manuscript. The Corresponding Author for this paper has been emended to be Juan Ausió instead of Ladan Kalani.

